# The relationships between positive coping style, resilience, and illness stigma in patients with Meige syndrome: a cross-sectional study

**DOI:** 10.3389/fpsyt.2025.1646084

**Published:** 2025-10-06

**Authors:** Meng Li, Mengtian Li, Ning Zhang, Linghan Zhou, Wenling Zhang, Kezhen Yang, Xiaoyu Yang, Shen Li, Yanhong Li, Feng Liu, Qiong Li, Junfan Wei, Chanchan Gao, Huawei Li

**Affiliations:** ^1^ Nursing Department, The Third People’s Hospital of Henan Province, Zhengzhou, China; ^2^ School of Nursing, Xinxiang Medical University, Xinxiang, China; ^3^ Anesthesiology Department, Henan Provincial People’s Hospital, Zhengzhou, China; ^4^ School of Rehabilitation Therapy, Henan Vocational College of Tuina, Luoyang, China; ^5^ School of Nursing, North Henan Medical University, Xinxiang, China; ^6^ The Seventh Clinical Medicine College of Guangzhou University of Chinese Medicine, Shenzhen, China; ^7^ Nursing Department, Nanjing Central Hospital, Nanjing, China; ^8^ Department of Oncology, School of Medicine, Zhongda Hospital, Southeast University, Nanjing, China

**Keywords:** Meige syndrome, illness stigma, cross-sectional study, influencing factors, resilience

## Abstract

**Objectives:**

The present study aimed to investigate the levels of illness stigma in patients with Meige syndrome and to determine the association of socio-demographic characteristics, positive coping style, and resilience with illness stigma in patients with Meige syndrome.

**Method:**

A cross-sectional survey using convenience sampling was conducted in four hospitals in China from March 2025 to May 2025. A total of 280 patients clinically diagnosed with Meige syndrome were recruited. A socio-demographic characteristic questionnaire, the Chinese version of the Stigma Scale for Chronic Illness, the Chinese version of the simplified coping style questionnaire, and the Chinese version of the 10-item Connor**–**Davidson resilience scale were used to perform this research. Statistical analyses were performed using SPSS 27.0.

**Result:**

A high level of illness stigma was observed among 280 participants, as reflected by a mean score of 90.40 ± 25.59. The present study identified a negative correlation between illness stigma, positive coping style, and resilience. Multiple linear regression analysis showed that living arrangement (β = −0.16, *p* = 0.007), self-care ability (β = 0.13, *p* = 0.026), positive coping style (β = −0.15, *p* = 0.012), and resilience (β = −0.18, *p* = 0.002) were significant factors associated with illness stigma in Meige syndrome patients.

**Conclusion:**

This study reveals that patients with Meige syndrome experience high levels of illness stigma, which is associated with factors including living arrangement, self-care ability, positive coping style, and resilience. Healthcare professionals should prioritize stigma reduction in individuals with Meige syndrome, particularly those living alone or with limited self-care abilities, through targeted psychological support that enhances positive coping and resilience.

## 1 Introduction

Meige syndrome, also known as cranial dystonia, is a rare neurological disorder characterized by involuntary and often severe muscle contractions that primarily affect the orbicularis oculi and lower facial muscles (1). These symptoms can lead to a range of functional impairments, such as problems with vision, speech, and eating, which can severely reduce a patient’s quality of life (2). In addition to its physical manifestations, however, Meige syndrome can have far-reaching psychological effects on its sufferers (3). The reason for this is that the external presentation of Meige syndrome often exposes patients to public misconceptions, social isolation, and discrimination (4).

Illness stigma refers to the negative social labeling and discriminatory perceptions suffered as a result of an illness or health condition (5). It is a social phenomenon in which people are ostracized, discriminated against, and excluded because they are perceived as “abnormal” or “inferior” because of a disease or condition (6). This phenomenon not only affects an individual’s social interactions but can also have far-reaching negative effects on his or her mental health, quality of life, and social integration (7). Studies have shown that illness stigma, both perceived and experienced, contributes to depressive symptoms, low self-esteem, and reduced treatment adherence in a wide range of chronic and neurological conditions (8, 9). In particular, Meige syndrome—although a relatively rare neurological disorder—presents a unique combination of physical symptoms, such as pronounced facial spasms and profound psychosocial challenges. Compared with other well-studied neurological conditions like Parkinson’s disease or epilepsy (10, 11), the facial characteristics of Meige syndrome draw more public attention, increasing patients’ exposure to social scrutiny and discrimination. Studies on focal dystonia have shown that visible motor symptoms are significantly associated with social withdrawal and mental distress (12). Moreover, Meige syndrome has been underrepresented in psychosocial research, despite growing qualitative evidence that these patients experience a high psychological burden (13). Therefore, focusing on Meige syndrome fills an important research gap and offers clinical insights for a particularly vulnerable population, as illness stigma in this context is not merely a reflection of social prejudice but a systemic health challenge that compromises patients’ physical and mental well-being, as well as treatment outcomes, through intertwined psychological, behavioral, and social mechanisms.

In order to face these challenges, patients may adopt different psychological mechanisms to cope with the stresses and social pressures associated with the disease (14). Coping style refers to the cognitive and behavioral strategies that individuals use to regulate emotions, reduce psychological burdens, or solve problems when confronted with stress, conflict, or adversity (15). It reflects both how a person appraises a stressful event and how he/she responds on an emotional and behavioral level (16). According to the transactional model of stress and coping (17), coping can be problem-focused (addressing the stressor directly) or emotion-focused (managing the emotional response), and it represents a relatively stable individual characteristic that can nonetheless be adapted to specific situations (18, 19). Prior studies have shown that patients with higher stigma are more likely to adopt negative coping strategies (20) and that coping plays a mediating role in the formation and alleviation of stigma: positive coping styles are protective, while negative coping may reinforce self-stigmatization (21). Importantly, multiple findings have shown that positive coping style demonstrates superior predictive power over negative coping style, particularly in terms of enhancing psychological resilience versus suppressing negative psychological outcomes (e.g., anxiety and fear) (22–24). Therefore, in the present investigation, we focused only on positive coping style. While coping style reflects the immediate responses that patients use to manage stress, long-term adaptation to illness may involve broader psychological resources that extend beyond these momentary strategies.

Resilience refers to an individual’s ability to effectively adapt to and recover from stress, trauma, adversity, tragedy, or significant threats (25). Unlike coping strategies, which reflect situational and short-term responses to stress, resilience represents a more enduring psychological resource that enables individuals to sustain adjustment over time. High levels of psychological resilience are associated with better psychological outcomes (26), improved social functioning (27), and enhanced positive coping style (28) in patients with a variety of diseases. There is also evidence that resilience is beneficial in reducing illness stigma in adolescent psychiatric patients (29). These findings indicate that resilience, together with coping style, may play a central role in shaping how patients experience illness stigma.

Although these associations have been established in other chronic and neurological conditions, little is known about whether similar mechanisms operate in Meige syndrome. So far, there is still a lack of research on illness stigma in patients with Meige syndrome. The specific relationship between resilience, coping strategies, and disease stigma among patients with Meige syndrome remains unclear, and further research is necessary. In light of these gaps, the present study aimed to investigate the level of illness stigma in patients with Meige syndrome and to explore the relationship between socio-demographic characteristics, resilience, positive coping style, and illness stigma. This will help to understand the psychological state of patients with Meige syndrome and provide valuable insights into future intervention strategies for patients with Meige syndrome.

## 2 Methods

### 2.1 Study design

The present study was implemented using the method of convenience sampling and a descriptive cross-sectional design (30), which has been widely used to investigate the status of psychological indicators in chronic illness patients. This study was performed according to the Strengthening the Reporting of Observational Studies in Epidemiology (STROBE) guidelines.

### 2.2 Participants

The sample size for this study was calculated according to the sample size calculation principles used in the Kendall cross-sectional survey, n = independent variable × (5 ~ 10) (31). Four scales with 10 independent variables were used in this study, which were derived from a socio-demographic characteristic questionnaire (including seven questions), the Chinese version of the Stigma Scale for Chronic Illness (including two dimensions), the Chinese version of the simplified coping style questionnaire (only positive coping style scale, including one dimension), and the Chinese version of the 10-item Connor–Davidson resilience scale (including one dimension). Considering the 20% invalid questionnaires, the minimum sample size required for this study was 68 to 138.

Participants were clinically diagnosed with Meige syndrome, aged 18 years or older, and capable of independently completing the questionnaire. Exclusion criteria included patients with medical records of severe mental disorders (such as major depressive disorder or schizophrenia) or cognitive impairments that hindered questionnaire comprehension or response.

### 2.3 Instruments

#### 2.3.1 Socio-demographic characteristic questionnaire

Based on a preliminary study and review, a socio-demographic characteristic questionnaire was designed to collect participants’ general information, including age, gender, residence, education level, marital status, living arrangement, and self-care ability.

#### 2.3.2 The Chinese version of Stigma Scale for Chronic Illness

The Stigma Scale for Chronic Illness (SSCI) is an instrument developed to measure stigma in chronic illness patients, with demonstrated reliability and validity. In 2009, Rao et al. (11) developed the SSCI based on Corrigan’s definition of stigma, which was initially used to measure the stigma experienced by patients with neurological diseases. This scale consists of 24 items grouped into two domains, namely, enacted stigma (11 items) and self-stigma (13 items). Enacted stigma assesses patients’ actual experiences of discrimination (e.g., people being unfriendly, making fun of, or staring at them), while self-stigma measures the internalization of negative stereotypes related to their condition (e.g., feeling different from others, avoiding new friendships to hide the illness, or blaming themselves for problems). Responses are rated on a 5-point Likert scale, where 1 indicates “never” and 5 indicates “always”. Total scores range from 24 to 120, with higher scores reflecting stronger levels of stigma. In a sample of 511 patients with neurological diseases, Rao et al. (11) demonstrated acceptable internal consistency, and the two domains were significantly correlated (*r* = 0.81). The Chinese version of the SSCI, translated by Deng et al. (32), demonstrated satisfactory reliability and validity and has been widely employed in diverse studies (33, 34). In the current study, the scale demonstrated excellent internal consistency, with a Cronbach’s α of 0.98.

#### 2.3.3 The Chinese Version of Simplified Coping Style Questionnaire

The Simplified Coping Style Questionnaire (SCSQ) was developed by Xie in 1998 (35), drawing on Lazarus and Folkman’s problem-focused and emotion-focused coping model (17). The simplified scale exhibited excellent reliability. It includes two dimensions: positive coping style (12 items, including asking relatives, friends, or classmates for advice and finding several different ways to solve the problem) and negative coping style (eight items, including reliance on others to solve problems). The scale is self-rated on a 4-point Likert scale with 0 = never and 3 = always. This study used only the Positive Coping Scale, so its score ranged from 0 to 36. The higher the total scores, the more likely the participants are to adopt positive coping style. In the current study, the scale demonstrated excellent internal consistency, with a Cronbach’s α of 0.79.

#### 2.3.4 The Chinese version of 10-item Connor–Davidson resilience scale

The 10-item Connor–Davidson resilience scale (CD-RISC-10) scale, originally developed by Campbell-Sills et al. (36), was employed to assess participants’ resilience. The Chinese version, which was translated and validated by Ye et al. (37), has shown satisfactory psychometric properties in terms of reliability and validity. Each item is rated on a 5-point Likert scale from 0 (almost never) to 4 (always). The total score ranged from 0 to 40, with higher total scores indicating greater resilience. In the current study, the scale demonstrated excellent internal consistency, with a Cronbach’s α of 0.95.

### 2.4 Data collection

A descriptive cross-sectional survey was conducted using convenience sampling. Data collection was conducted from March 2025 to May 2025 in four tertiary hospitals in Henan Province. During the study period (March to May 2025), all patients who met the inclusion criteria and were diagnosed with Meige syndrome at the participating hospitals were approached for participation. Recruitment was conducted through outpatient clinics and inpatient wards. Only those who voluntarily agreed to participate and provided written informed consent were enrolled in the study. Researchers personally distributed paper questionnaires to participants in quiet, private settings (e.g., conference rooms or consultation areas) to ensure confidentiality. The questionnaire comprised four components: a socio-demographic characteristic questionnaire, the SSCI, the positive coping style scale of SCSQ, and the CD-RISC-10. All responses were anonymous, with no collection of personally identifiable information (e.g., names and addresses). After completion, trained staff verified the integrity of each questionnaire. Two researchers independently managed paper questionnaire collection and data entry into a computer system, cross-checking for discrepancies and resolving any inconsistencies promptly. Participants were recruited from outpatient clinics and inpatient wards of the study sites. Eligible participants were provided with verbal and written explanations of the study objectives, their rights as participants, and the confidentiality measures in place. Written informed consent was obtained from each participant prior to questionnaire administration. Ethical approval for the study was granted by the institutional review board before data collection, and all data were securely stored and accessible only to the research team.

### 2.5 Data analysis

The data were analyzed using SPSS Statistics version 27.0 (IBM Corporation, Armonk, NY, USA). Normality of continuous data was assessed using the Kolmogorov–Smirnov test. Since all data were normally distributed, continuous data were presented as mean (standard deviation). Outliers were checked by boxplots and descriptive statistics; extreme values were examined but retained if clinically plausible. Standardization procedures (e.g., z-scores) were not performed because analyses were based on validated raw scale scores. Categorical data were presented as percentages (38). To examine whether illness stigma in patients with Meige syndrome varied across different socio-demographic variables, independent-samples t-tests and one-way ANOVA were conducted (39). To assess the relationships among SSCI scores, positive coping style scores, and CD-RISC-10 scores, Pearson’s correlation analysis was conducted. Multi-collinearity was evaluated through the calculation of tolerance and the variance inflation factor (VIF). The absence of multi-collinearity was confirmed when tolerance exceeded 0.1 and the VIF remained below 10. Variables found to be statistically significant in the t-test, ANOVA, and Pearson’s correlation analyses were identified as potential predictors for inclusion in the regression analysis. Finally, multiple linear regression was conducted with potential predictors as independent variables and illness stigma as the dependent variable to confirm the relationship between Meige syndrome patients’ socio-demographic characteristics, resilience, and coping style. In addition, an additional *post-hoc* power analysis for each of the two phases was performed. A two-tailed *p* < 0.05 was considered statistically significant.

### 2.6 Ethical considerations

Ethical approval for this study was obtained from the Third People’s Hospital of Henan Province in Zhengzhou, China (Ethical Review No. 2025SZSYLCYJ0302), based on the principles of the Declaration of Helsinki. All eligible participants were informed of the study and its ethical principles (e.g., voluntary participation, withdrawal, anonymity, and confidentiality). All participants were fully informed about the study objectives, procedures, rights, and confidentiality protections, and all provided written informed consent prior to participation. After completing the survey, participants were thanked and provided with general health education materials; no individual results were disclosed to ensure confidentiality. All data were securely stored and accessible only to the research team.

## 3 Results

### 3.1 Socio-demographic characteristics of participants and comparison of different variables on stigma in Meige syndrome patients

A total of 400 participants were invited to participate in this study, and 280 valid questionnaires were collected, resulting in an effective response rate of 70.0%. The results of the study found that the total illness stigma score of the 280 participants was 90.40 ± 25.59. Among the 280 participants, the gender distribution was relatively balanced, with two-thirds of the participants residing in the city. In terms of education, about two-thirds had completed at least junior high school. Most participants were married, and more than half lived alone. In terms of self-care ability, the largest proportion of patients were partially dependent, with fewer being fully independent or fully dependent. t-Tests and ANOVA revealed that illness stigma in Meige syndrome patients showed statistically significant differences in residence, living arrangement, and self-care ability (*p* < 0.05). Specifically, patients residing in city areas, those living alone, and those who are fully dependent exhibited higher illness stigma scores. Conversely, no statistically significant differences in stigma were observed based on gender, education level, or marital status (*p* > 0.05). See **Table 1** for details.

**Table 1:** Socio-demographic characteristics and comparison of participants’ different variables regarding illness stigma in Meige syndrome (N = 280).

Variables	Categories	N	%	*M* ± *SD*	t/F	*p*
Age		280	100.0	60.67 ± 7.38	−0.017	0.779
Gender	Male	126	45.0	90.92 ± 26.11	0.308	0.758
	Female	154	55.0	89.96 ± 25.22		
Residence	City	179	63.9	92.73 ± 24.36	1.986	0.048
	Village	101	36.1	86.24 ± 27.25		
Education level	Below primary school	19	6.8	83.74 ± 29.00	0.467	0.587
	Primary school	82	29.3	87.96 ± 25.02		
	Junior high school	65	23.2	85.89 ± 27.84		
	Senior high school	65	23.2	91.45 ± 24.02		
	Junior college’s degree	29	10.4	87.03 ± 26.50		
	Bachelor’s degree	11	3.9	81.55 ± 37.48		
	Postgraduate degree	9	3.2	91.00 ± 23.51		
Marital status	Unmarried	1	0.4	92.00	0.056	0.955
	Married	251	89.6	87.76 ± 26.29		
	Divorced	17	6.1	89.00 ± 27.66		
	Widowed	11	3.9	85.18 ± 27.01		
Living arrangement	Living alone	166	59.3	94.86 ± 23.46	3.500	0.001
	Living with others	114	40.7	83.89 ± 27.21		
Self-care ability	Fully independent	57	20.4	75.54 ± 28.63	7.115	0.001
	Partially dependent	148	52.9	90.41 ± 24.56		
	Fully dependent	75	26.8	91.80 ± 25.24		

Pearson’s correlation analysis was used for continuous variables. Independent-samples t-tests were used for binary variables; one-way ANOVA was used for multi-category variables.

*M*, mean; *SD*, standard deviation.

### 3.2 Correlation analysis of stigma, coping style, and psychological resilience

In this study, illness stigma was found to be correlated with positive coping style and resilience. Specifically, the total illness stigma score showed a negative correlation with the total score of positive coping style (*r* = −0.225, *p* < 0.001), indicating that higher illness stigma was associated with less frequent use of positive coping style. Similarly, the total stigma score was significantly negatively correlated with resilience (*r* = −0.176, *p* = 0.003), suggesting that higher stigma was associated with lower levels of resilience. However, positive coping style scores were positively correlated with resilience (*r* = 0.163, *p* < 0.001), indicating that individuals who employed positive coping strategies tended to have higher levels of resilience. See **Table 2** for details.

**Table 2:** Pearson’s correlation of positive coping style, resilience, and stigma in patients with Meige syndrome.

Variables	Positive coping style (total score)	Resilience (total score)	Illness stigma (total score)
Positive coping style (total score)	1		
Resilience (total score)	0.163**	1	
Illness stigma (total score)	−0.225**	−0.176**	1

Correlations were assessed using Pearson’s correlation analysis.

**p < 0.01.

### 3.3 Multiple linear regression analysis of factors associated with illness stigma in Meige syndrome patients

Multi-collinearity among the independent variables was assessed to verify the basic assumptions of the regression model. Given that the VIF was 10 or less and the tolerance was 0.1 or above, no significant multi-collinearity issues were identified. The Durbin–Watson statistic was 1.818, close to 2.0, indicating no autocorrelation and suggesting that the error terms were independent of each other. Based on univariate analysis of t-test, ANOVA, and Pearson’s analysis, variables with statistical significance (*p* < 0.05) were selected as independent variables, with illness stigma as the dependent variable for multiple linear regression analysis. As shown in **Table 3**, the model explained a significant proportion of the variance in stigma among Meige syndrome patients. The results indicated that living arrangement (β = −0.16, *p* = 0.007), self-care ability (β = 0.13, *p* = 0.026), positive coping style (β = −0.15, *p* = 0.012), and resilience (β = −0.18, *p* = 0.002) were significant factors associated with illness stigma in Meige syndrome patients.

**Table 3:** Multiple linear regression analysis for factors associated with illness stigma in patients with Meige syndrome.

Variables	*B*	SE	β	t	*p*	95% CI		Tolerance	VIF
						Lower	Upper		
Constant	114.67	8.41		13.42	0.000				
Residence	−5.71	3.04	−0.11	−1.88	0.061	−11.69	0.26	0.970	1.031
Living arrangement	−8.16	3.02	−0.16	−2.70	0.007	−14.11	−2.22	0.937	1.067
Self-care ability	4.85	2.17	0.13	2.24	0.026	0.53	9.11	0.940	1.063
Positive coping style	−0.53	0.21	−0.15	−2.53	0.012	−0.95	−0.12	0.963	1.038
Resilience	−0.37	0.12	−0.18	−3.07	0.002	−0.61	−0.13	0.970	1.031

*R* = 0.367, *R* ^2^ = 0.134, adjusted *R* ^2^ = 0.119.

*B*, partial regression coefficient; SE, standard error; β, standardized regression coefficient; 95% CI, 95% confidence interval for *B*; VIF, variance inflation factor.

## 4 Discussion

This study investigated illness stigma in patients with Meige syndrome and examined its associations with socio-demographic characteristics, coping style, and resilience. The findings revealed three main points. First, patients with Meige syndrome experienced a markedly high level of illness stigma, with mean scores significantly exceeding those reported in other chronic and neurological conditions. Second, socio-demographic variables, particularly living alone and low self-care ability, were associated with higher levels of stigma. Third, psychological resources such as positive coping style and resilience were negatively associated with stigma, suggesting their protective role. To better understand these findings, the following sections discuss each aspect in detail.

Our study revealed that patients with Meige syndrome experienced a high level of illness stigma, with a mean score of 90.40 ± 25.59. This value is significantly higher than those reported in previous studies of chronic disease populations (40). Moreover, in research involving multiple neurological disorders such as epilepsy, multiple sclerosis, Parkinson’s disease, and stroke, the average stigma score was 42.7 ± 19.7 (11), which is less than half of the mean observed in our sample. These comparisons highlight that individuals with Meige syndrome are subject to exceptionally high levels of stigmatization, likely due to the visibility and uncontrollability of their facial and motor symptoms. Such manifestations may draw unwanted social attention, foster misinterpretations, and intensify experiences of discrimination, thereby exacerbating internalized stigma. This finding underscores the urgent need for greater clinical and social interventions to reduce stigma in this patient group, as well as the importance of public education to improve awareness and reduce misconceptions about Meige syndrome.

Our findings showed that the patients’ illness stigma score for living alone was significantly higher than that of patients living with family. On the one side, the reason may be that living with family members offers a close‐knit, supportive community that could reduce stigma. One possible explanation is that cohabiting with family provides a supportive environment that buffers against social alienation and reduces perceived stigma, a notion supported by studies showing that stronger family and social bonds are associated with lower stigmatization (41, 42). On the other side, living alone has been associated with poor mental health conditions (43–45). Stahl et al. (1) found that living alone was associated with elevated levels of depressive symptoms compared to living with a family member. Similar findings were also noted in the elderly population (46). In a qualitative meta-analysis, Hu et al. (47) found that older people living alone had a higher risk of depression than those not living alone (47). Consistent with our results, the relationship between living alone and anxiety has also been discussed in previous research; for example, Hunt et al. (48) and Yu et al. (49) found that people living alone had a significantly higher risk of generalized anxiety disorder than those living with their families. These findings suggest that social support—especially from family members—plays a crucial role in protecting patients with Meige syndrome from the psychological burden of stigma. Interventions that strengthen family involvement and foster broader social support networks may therefore be effective strategies to mitigate stigma in this population.

Our study found that higher self-care ability is associated with lower stigma. Consistent with our findings, Asuka Katoo et al. (50) demonstrated in a cohort of 209 type 2 diabetes patients that self-stigma was significantly and inversely associated with self-care behaviors (β = −0.23, *p* = 0.001), independent of self-efficacy and depressive symptoms. A possible explanation is that self-care enhances patients’ sense of autonomy and competence, which may counteract internalized stigma. Conversely, diminished self-care capacity can erode self-efficacy, increase dependence on others, and heighten vulnerability to stigmatization, a pattern also observed in disability-related research (51, 52). These findings suggest that self-care capability may reduce stigma by fostering autonomy and competence, whereas loss of independence can erode self-worth and increase vulnerability to stigmatization. Moreover, inadequate self-care often manifests in visible neglect, which can trigger negative social judgments and marginalization (53, 54). Although the correlation observed in our study was relatively weak (|*r*| < 0.3) and should be interpreted with caution, it nevertheless suggests that strengthening patients’ ability to manage their daily health could serve as a practical strategy for stigma reduction. Interventions that foster self-care skills and incorporate cognitive reframing or acceptance-based approaches may help patients maintain autonomy, mitigate feelings of worthlessness, and better cope with the visible symptoms of Meige syndrome (55).

We also observed a significant negative correlation between the total positive coping style score and the illness stigma score among patients with Meige syndrome (*r* = −0.225, *p* < 0.01). Although the effect size was relatively small (|*r*| < 0.3) and should be interpreted with caution, the finding aligns with coping theory, which highlights the protective role of adaptive coping strategies in reducing stress and stigma (17). Positive coping behaviors—such as seeking social support, reframing the meaning of illness, regulating emotions, and adopting problem-solving orientations—may help patients reinterpret their condition as a manageable challenge rather than a personal failure, thereby reducing internalized stigma (21). In addition, positive coping tends to promote social engagement and help-seeking, counteracting the avoidance and isolation that often characterize stigmatized individuals. Li et al. (13) pointed out that those Meige syndrome patients who actively sought external support perceived significantly lower levels of stigmatization. Nevertheless, cultivating positive coping habits may still help patients mobilize internal resources and enhance their self-efficacy in dealing with disease-related challenges, thereby contributing to the development of a more resilient psychological defense system.

In addition, a significant negative correlation was observed between resilience and illness stigma (*r* = −0.176, *p* < 0.01). Although the effect size was weak (|*r*| < 0.3) and should be interpreted with caution in terms of clinical significance, the direction of the association suggests that resilience may serve as a protective factor against stigmatization. This interpretation is consistent with previous studies showing that resilience fosters psychological adjustment and buffers stigma (24, 28). Other research has shown that individuals with higher resilience are often better able to manage emotions, maintain a positive mindset, and demonstrate greater psychological stability in the face of adversity (56, 57). Importantly, recent research also suggests a bidirectional link, whereby perceived stigma can erode resilience and, in turn, amplify social anxiety symptoms (58, 59). Such findings point to an interactive process between stigma and resilience that may exacerbate psychosocial vulnerability in patients with visible conditions like Meige syndrome. Clinically, these findings highlight the value of resilience as a protective factor, suggesting that interventions aiming to foster resilience should be prioritized to improve psychological well-being and reduce stigma in patients with Meige syndrome.

In addition, individuals with higher resilience typically possess more mature cognitive and physiological regulatory mechanisms, which enhance their capacity to adopt positive coping strategies when facing stress and challenges. For example, Yu Wu et al. (60) found that undergraduates with greater resilience and better emotion regulation demonstrated more flexible and adaptive coping styles. In the context of Meige syndrome, this interplay between resilience and coping may be especially important: stronger resilience facilitates positive coping, which in turn alleviates the physical and psychological burden of the disease and improves overall quality of life. This reciprocal relationship suggests a potential “virtuous cycle”, where resilience and coping mutually reinforce each other to buffer against stigma. These findings imply that psychological interventions should not only aim to enhance resilience but also incorporate structured coping skills training, enabling patients to build a more solid psychological defense system and adapt more effectively to the challenges of their illness and daily life.

In conclusion, this study found that people with Meige syndrome experience high levels of stigmatization. It comprehensively revealed that living alone, low self-care ability, and poor positive coping style are risk factors exacerbating illness stigma among Meige syndrome patients, while resilience and positive coping style act as protective factors. The significant correlations uncovered positive coping with psychological resilience, and both coping style and resilience with stigma underscored the intricate interplay of these factors. These findings highlight the need for multi-dimensional clinical interventions that focus on fostering positive coping mechanisms, enhancing psychological resilience, and addressing the unique social pressures faced by patients. By targeting these aspects, healthcare providers can effectively reduce stigma, improve mental well-being, and facilitate better adaptation to the challenges posed by Meige syndrome, ultimately promoting a more positive quality of life for patients.

## 5 Limitations

Despite efforts to enhance the methodological rigor of this study, several limitations should be noted. First, the demographic representativeness of the sample is limited, as participants were primarily drawn from specific geographic regions, potentially restricting the generalizability of the findings. Second, the cross-sectional design limited causal inferences regarding the associations among stigma, psychological resilience, and positive coping style. Third, the study focused predominantly on individual-level psychological variables, without extensive examination of broader contextual factors such as community support networks or healthcare accessibility.

## 6 Conclusion

This study reveals that patients with Meige syndrome experience high levels of illness stigma, which is associated with factors including living arrangement, self-care ability, positive coping style, and resilience. The findings indicated that patients who demonstrated higher resilience and adopted positive coping strategies experienced lower levels of perceived stigma, highlighting the need for targeted interventions that address the specific challenges faced by Meige syndrome patients, particularly in terms of reducing stigma and enhancing psychological resilience. Healthcare professionals should focus on the level of stigma in people with Meige syndrome who live alone or have low self-care abilities. Targeted psychological support measures focusing on positive coping style and resilience should be implemented for all patients with Meige syndrome. Future research should consider longitudinal designs to capture temporal changes in stigma and resilience and investigate the influence of external support systems in mitigating stigma among individuals with Meige syndrome.

## Data Availability

The raw data supporting the conclusions of this article will be made available by the authors, without undue reservation.
